# Supplementary material for: Disorder-induced enhancement of lithium-ion transport in solid-state electrolytes
Source: Nat Commun. 2025 Jan 26;16:1057. doi: 10.1038/s41467-025-56322-x (PMC11770192; doi:10.1038/s41467-025-56322-x)
Supplement: Supplementary file 1 — Supplementary Information [file 41467_2025_56322_MOESM1_ESM.pdf]

SUPPLEMENTARY INFORMATION for

Disorder-induced enhancement of lithium-ion transport in solid-state electrolytes

Zhimin Chen[1], Tao Du[1,2*], N. M. Anoop Krishnan[3], Yuanzheng Yue[1], Morten M. Smedskjaer[1,*]

[1] *Department of Chemistry and Bioscience, Aalborg University, Aalborg East 9220, Denmark*

[2] *Department of Applied Physics, The Hong Kong Polytechnic University, Kowloon, Hong Kong, 999077, China*

[3] *Department of Civil Engineering, Indian Institute of Technology Delhi, New Delhi 110016, India*

* *Corresponding authors. E-mail: tao.du@polyu.edu.hk (T.D.), mos@bio.aau.dk (M.M.S.)*

Supplementary Figures

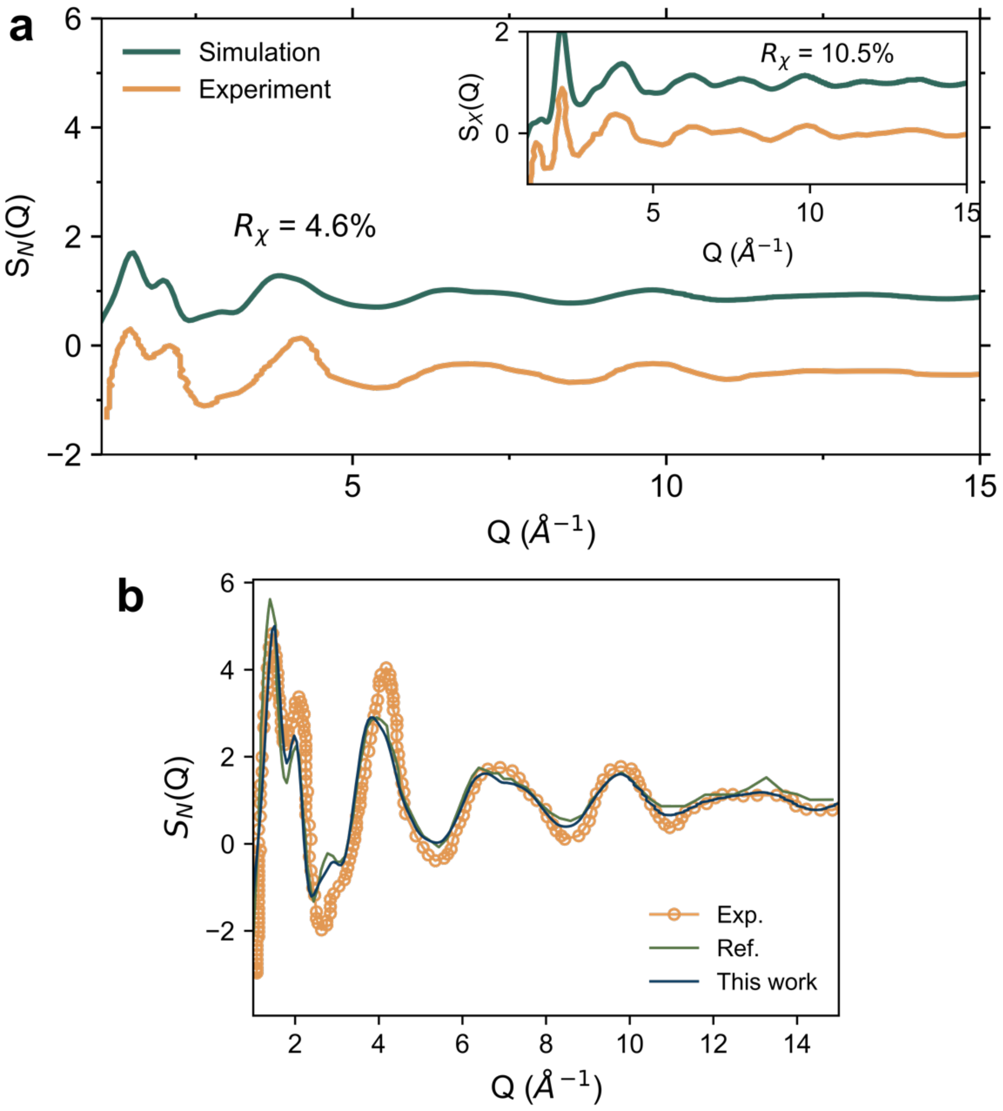

Fig. S1. a Neutron structure factor, $S_N(Q)$, comparison from MD simulations (using the present MLIP) and neutron scattering experiments[1] for glassy Li_3PS_4. The inset shows the X-ray structure factor, $S_X(Q)$, comparison with X-ray scattering experiments[1]. **b** Comparison of simulated and experimental neutron structure factor $S_N(Q)$. Experimental and other MLIP-calculated $S_N(Q)$ were obtained from Refs[1,2]. Source data are provided as a Source Data file.

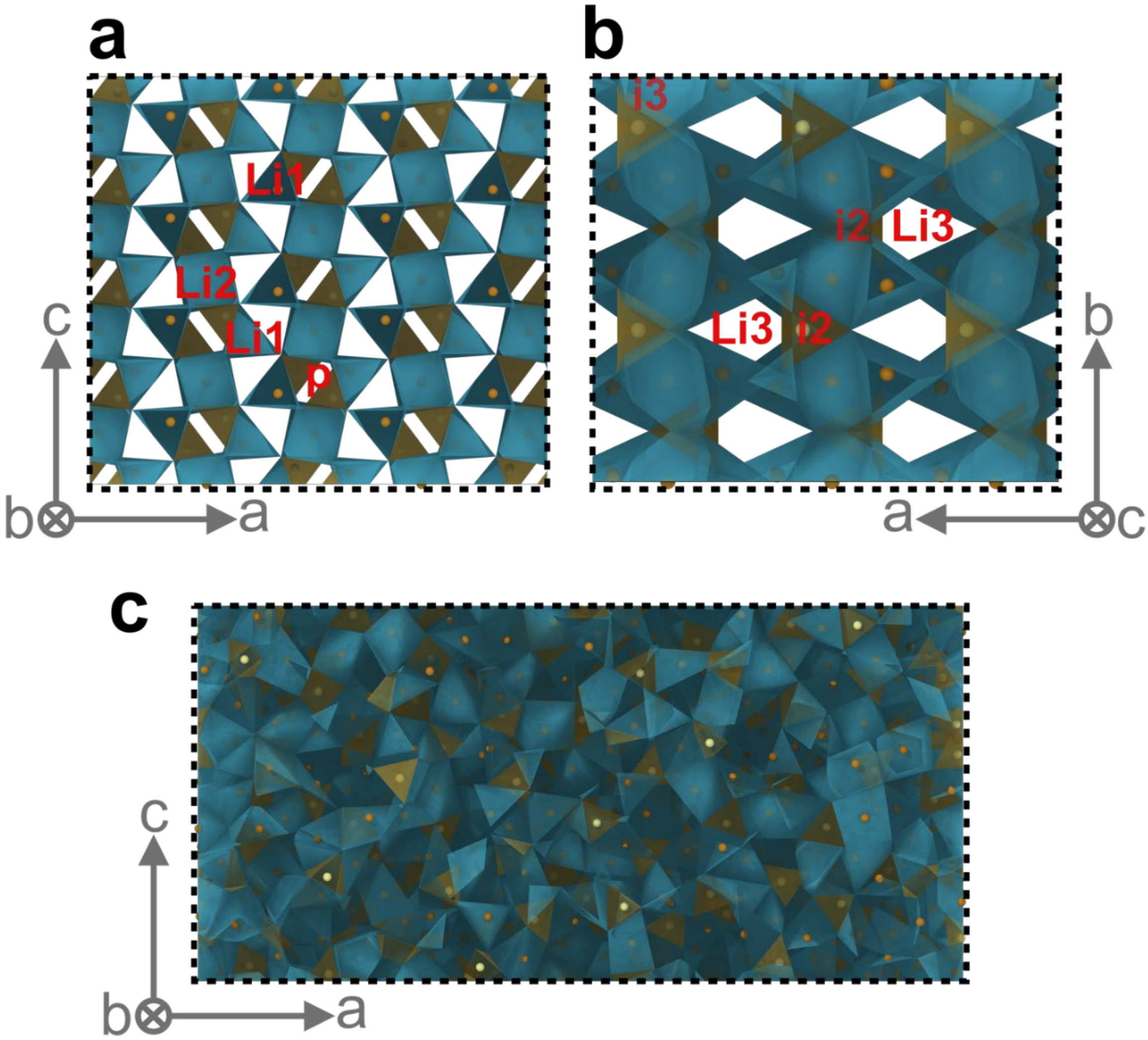

Fig. S2. Atomic snapshots of β-Li_3PS_4 (**a,b**) and glassy Li_3PS_4 (**c**) electrolyte configurations. Li1 and Li2 are lithium sites located at the centers of Li-S tetrahedra and octahedra, respectively. Li3 is a tetrahedral interstitial site, while i2 and i3 are octahedral interstitial sites. The snapshots are captured from localized regions within the final relaxed configurations in the MD simulation using the present MLIP.

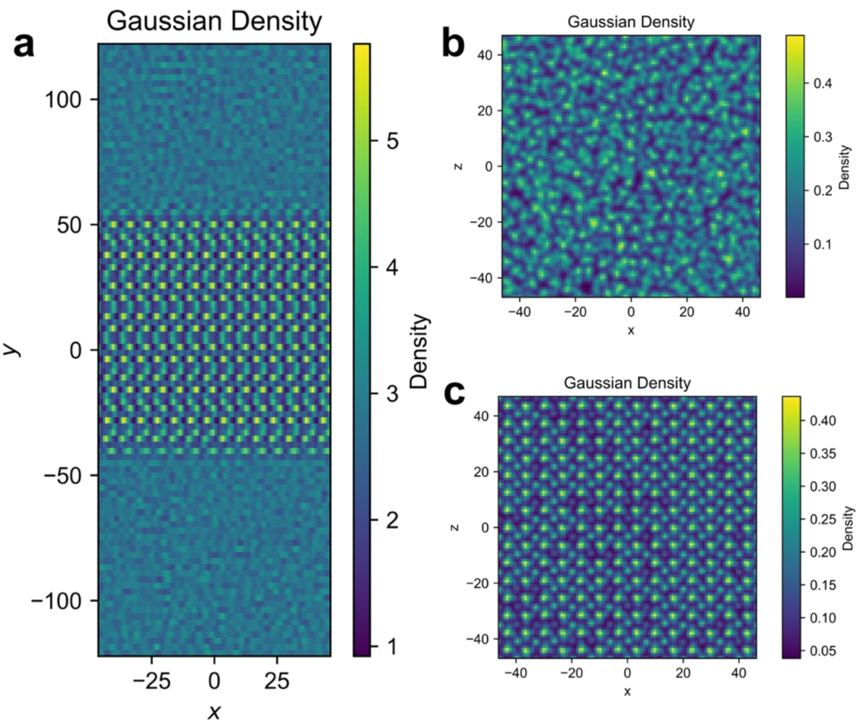

Fig. S3. Gaussian density distribution projections of P and S atoms in the 2D plane for glass-ceramic Li_3PS_4 (**a**), glassy Li_3PS_4 (**b**), and β-Li_3PS_4 (**c**) electrolytes.

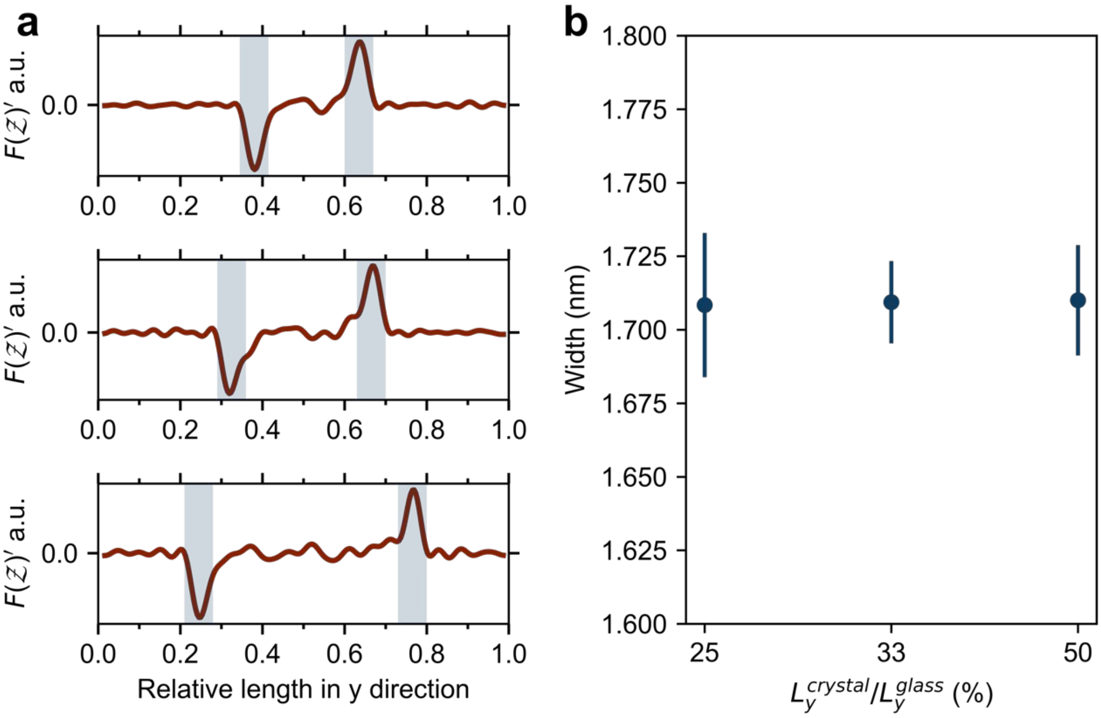

Fig. S4. a Amorphization distribution of the constructed glass-ceramic with varying crystalline content along the *y*-axis. The gray span highlights the internal interface between the ordered and disordered phases. **b** Width of interface area. Source data are provided as a Source Data file.

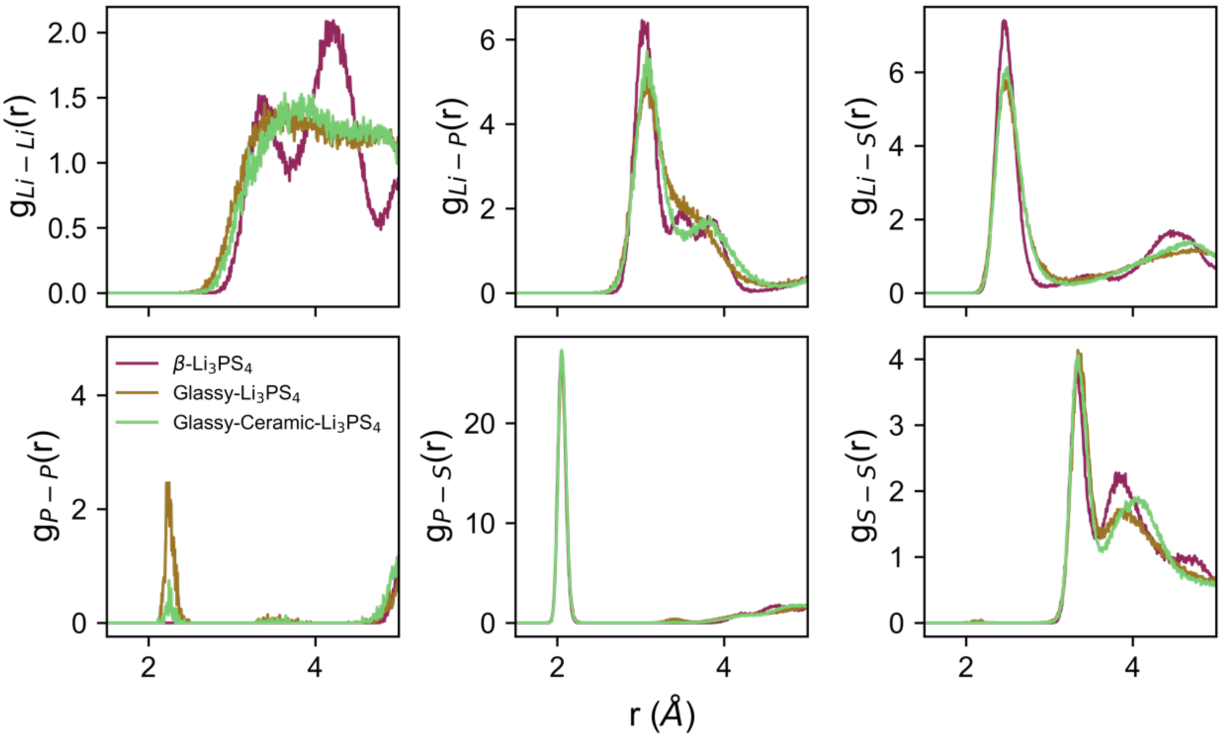

Fig. S5. Partial radial distribution functions $g_{ij}(r)$ of all atomic pairs in the simulated glassy, β-, and glass-ceramic Li_3PS_4 electrolytes using the present MLIP. Source data are provided as a Source Data file.

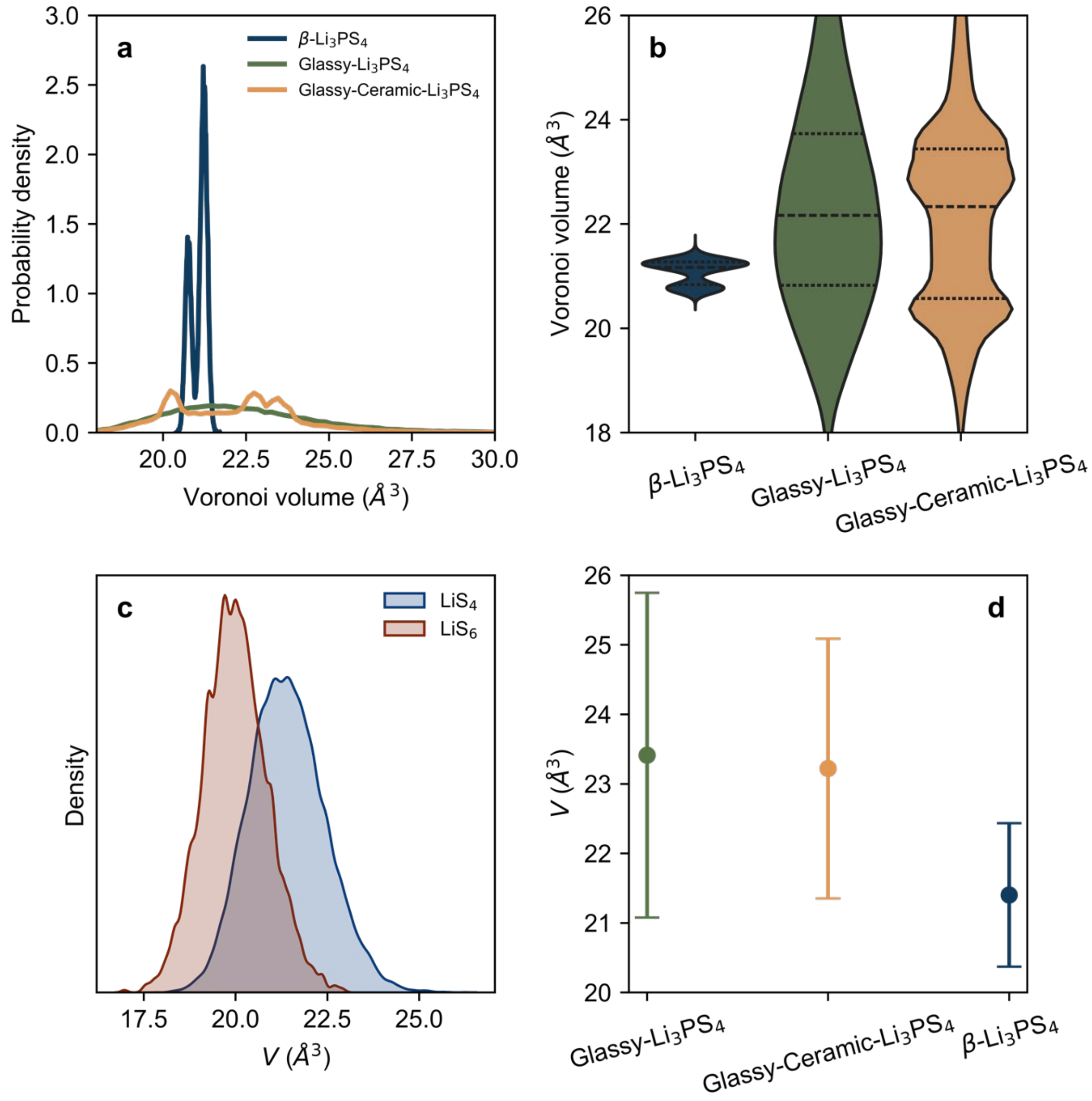

Fig. S6. Voronoi volume of lithium atoms in simulated Li_3PS_4 electrolytes using the MLIP: (a) density distribution and (b) violin plot. (c) Voronoi volume distribution of four-fold and six-fold coordination lithium atoms. (d) Averaged volume of four-fold coordinated lithium atoms in simulated glassy Li_3PS_4, β-Li_3PS_4, and glass-ceramic Li_3PS_4 electrolytes. Source data are provided as a Source Data file.

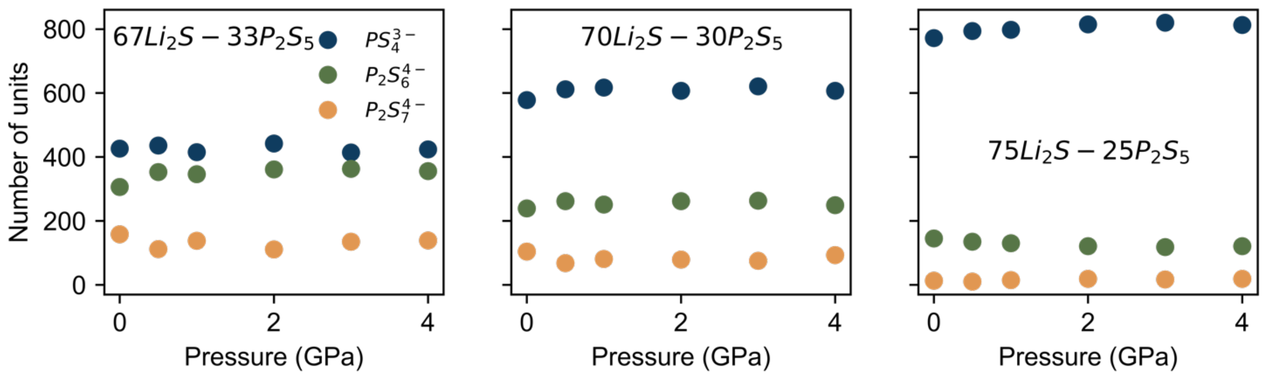

Fig. S7. Number of P-S units in three different Li_2S-P_2S_5 glassy electrolytes at different pressure. Source data are provided as a Source Data file.

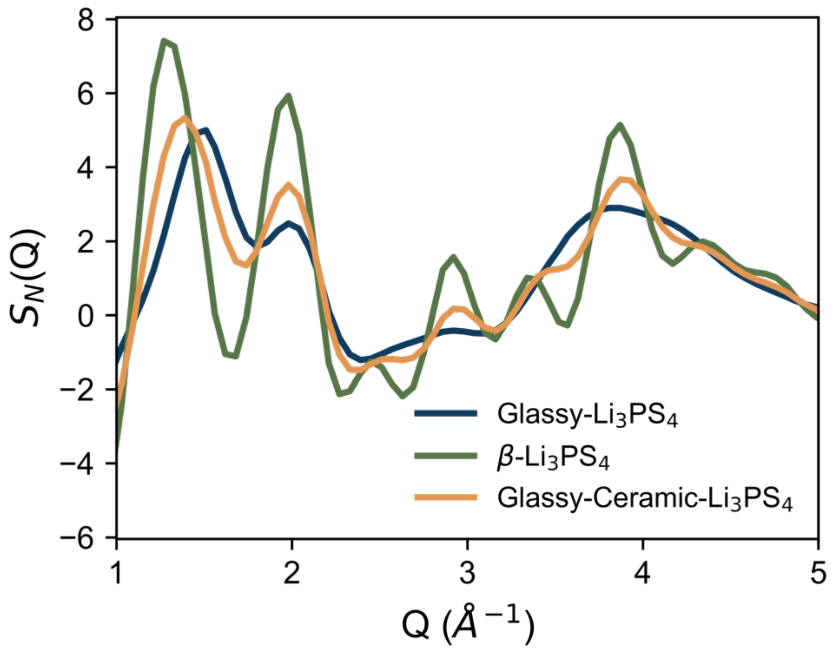

Fig. S8 Neutron structure factor $S_N(Q)$ of simulated glassy Li_3PS_4, β-Li_3PS_4, and glass-ceramic Li_3PS_4 electrolytes using the present MLP. Source data are provided as a Source Data file.

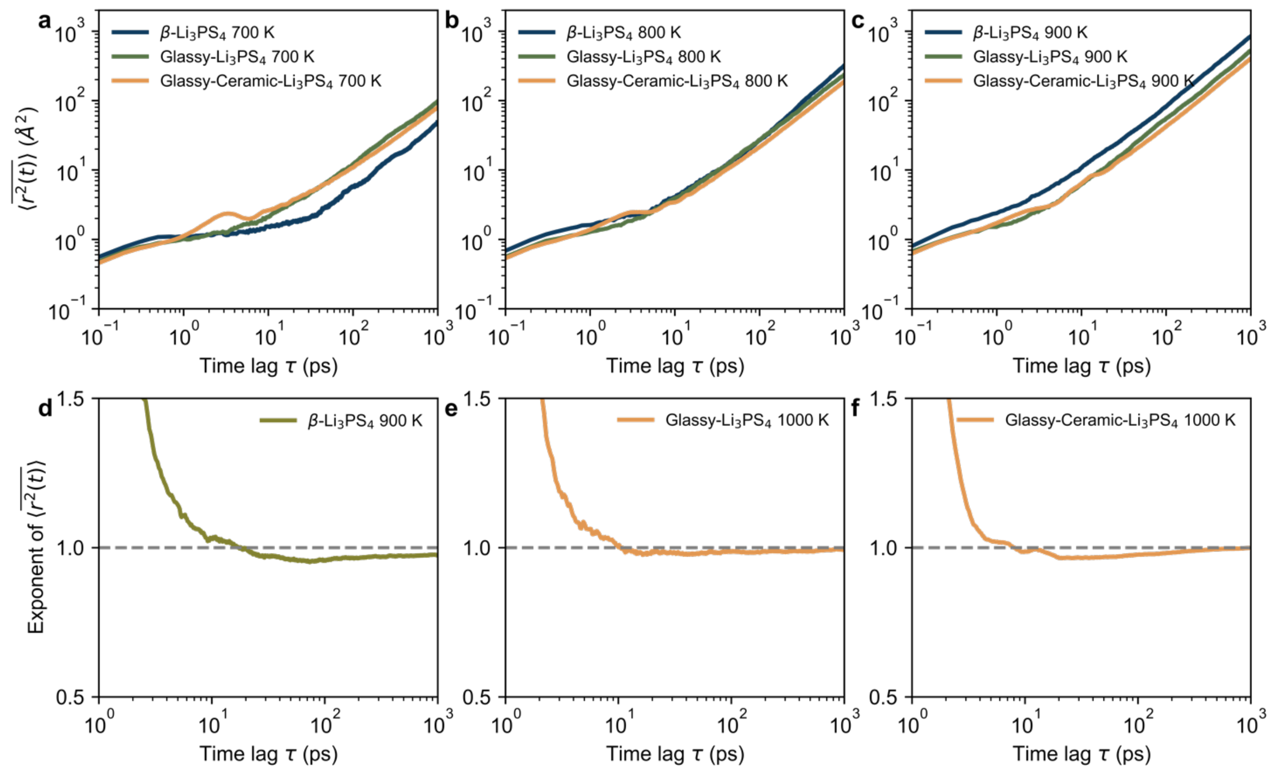

Fig. S9. a-c Mean squared displacement of Li_3PS_4 electrolytes at different temperature. **d-f** Exponent of mean squared displacement. The horizontal dashed lines represent the Fickian limit t^1. Source data are provided as a Source Data file.

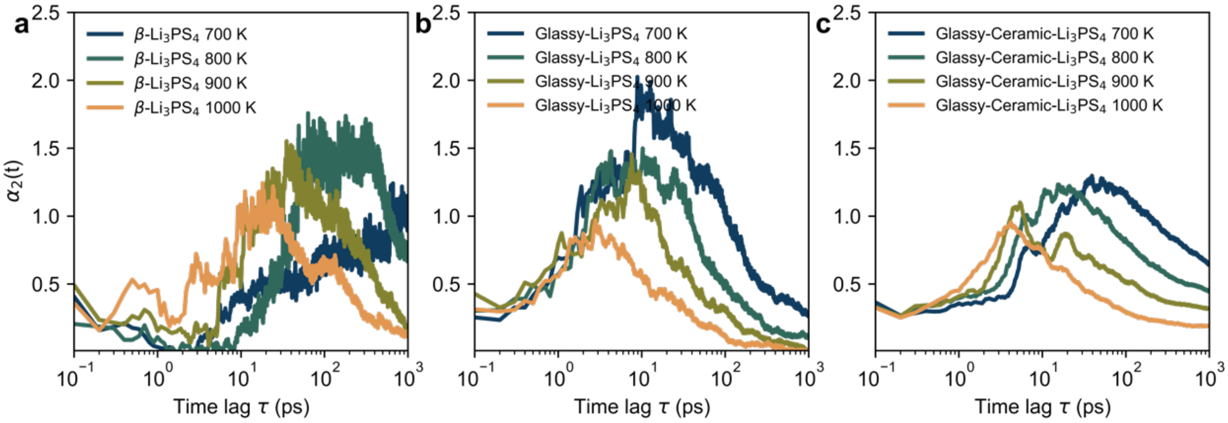

Fig. S10. Non-Gaussian parameter of **a** β- Li_3PS_4, **b** glassy Li_3PS_4, and **c** glass-ceramic Li_3PS_4 electrolytes Li_3PS_4 electrolytes at different temperatures (from 700 K to 1000 K). Source data are provided as a Source Data file.

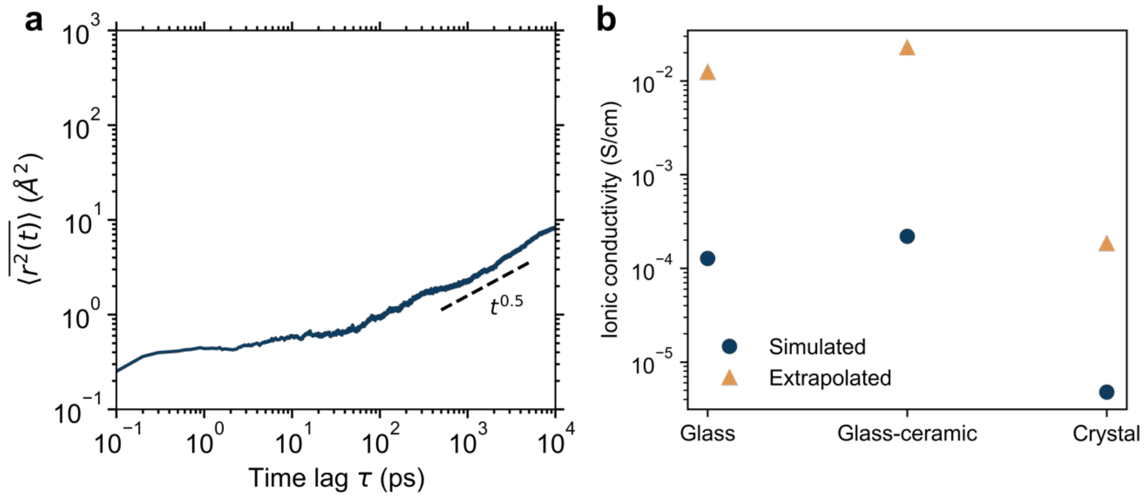

Fig. S11. **a** Mean squared displacement (MSD, $\overline{\langle r^2(t) \rangle}$) of Li_3PS_4 glass at 300 K. **b** Room temperature ionic conductivity obtained from long-time scale MD simulations at 300 K and extrapolation of Arrhenius fit. Source data are provided as a Source Data file.

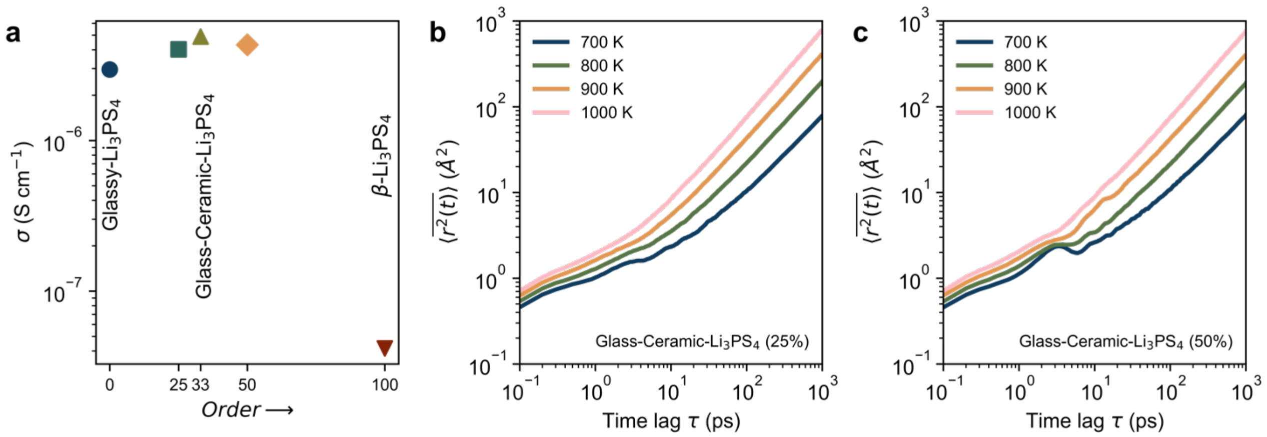

Fig. S12. a Room temperature ionic conductivity of glassy Li_3PS_4, glass-ceramic Li_3PS_4, and β-Li_3PS_4 electrolytes. The horizontal axis represents the fraction (%) of crystalline content. **b,c** Mean squared displacement of glass-ceramic Li_3PS_4 electrolytes with crystalline contents of **b** 25% and **c** 50%. Source data are provided as a Source Data file.

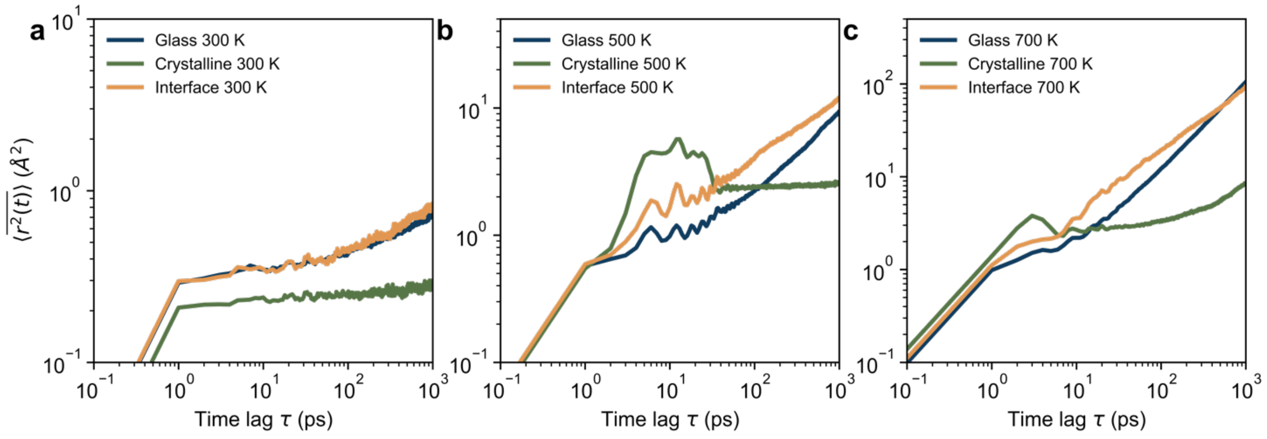

Fig. S13. Mean squared displacement of glass, crystalline, and interface phases within the simulated glass-ceramic Li_3PS_4 electrolytes at different temperatures (**a** 300 K, **b** 500 K, **c** 700 K). Source data are provided as a Source Data file.

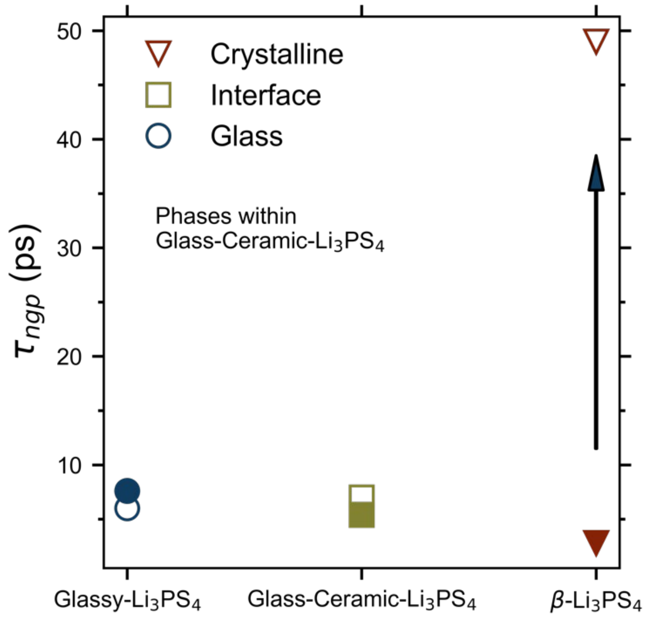

Fig. S14. Non-Gaussian parameter (NGP) peak times, τ_{ngp} for crystalline, interface, and glassy phases in glass-ceramic Li_3PS_4 (hollow symbol). Solid symbols represent the value of τ_{ngp} for glassy Li_3PS_4, β-Li_3PS_4, and glass-ceramic Li_3PS_4 electrolytes. Source data are provided as a Source Data file.

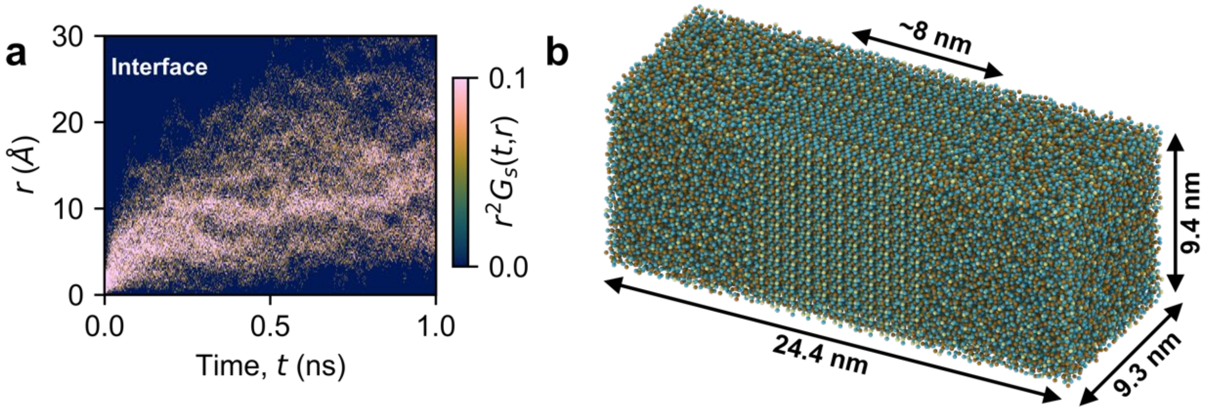

Fig. S15. a Self-part van Hove correlation function of interfacial phases within glass-ceramic Li_3PS_4 at 900 K. **b** Atomic snapshot of simulated glass-ceramic Li_3PS_4, with annotated dimensions representing the sizes of the crystalline and glassy phases.

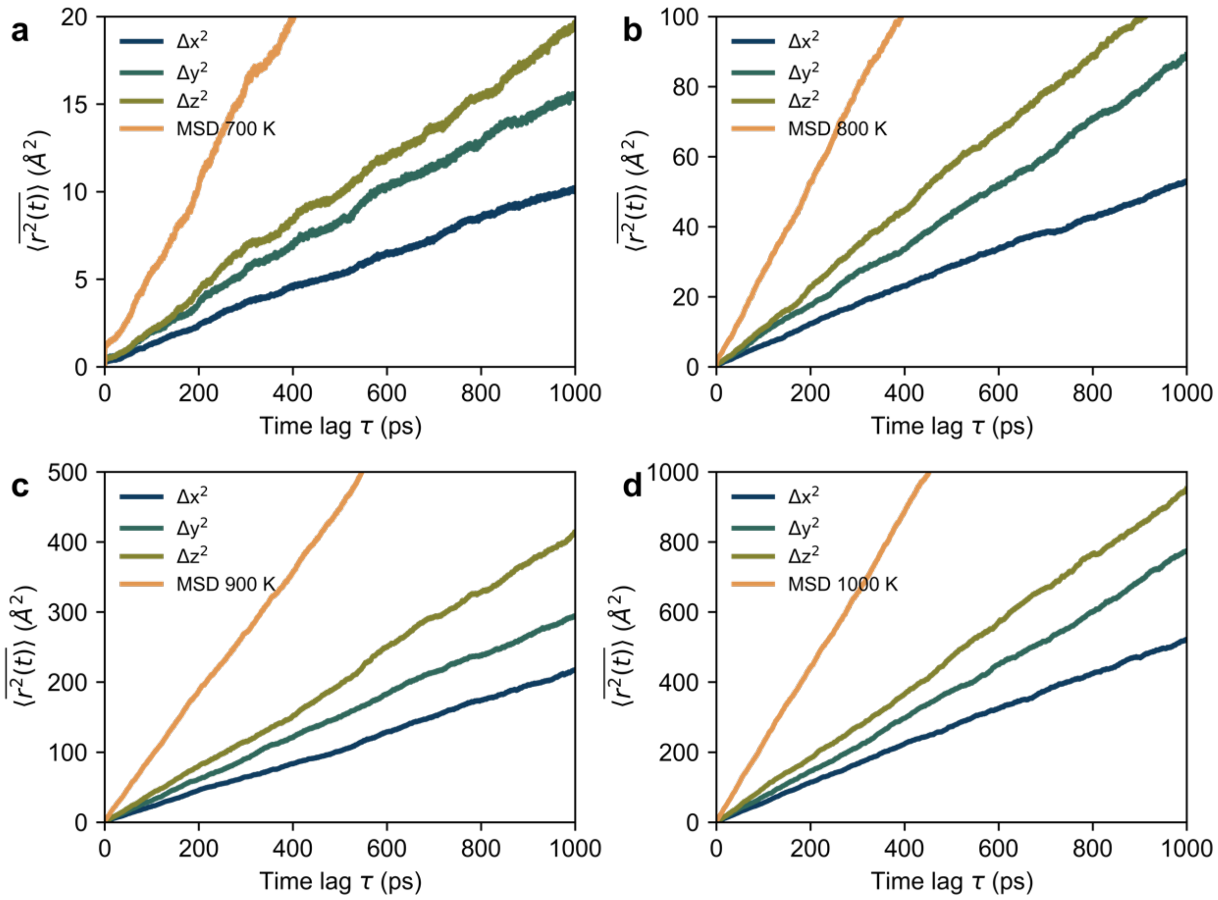

Fig. S16. Components of mean squared displacement of β- Li_3PS_4 in different directions, where panels **a** to **d** represent mean squared displacement at temperatures ranging from 700 K to 1000 K. Source data are provided as a Source Data file.

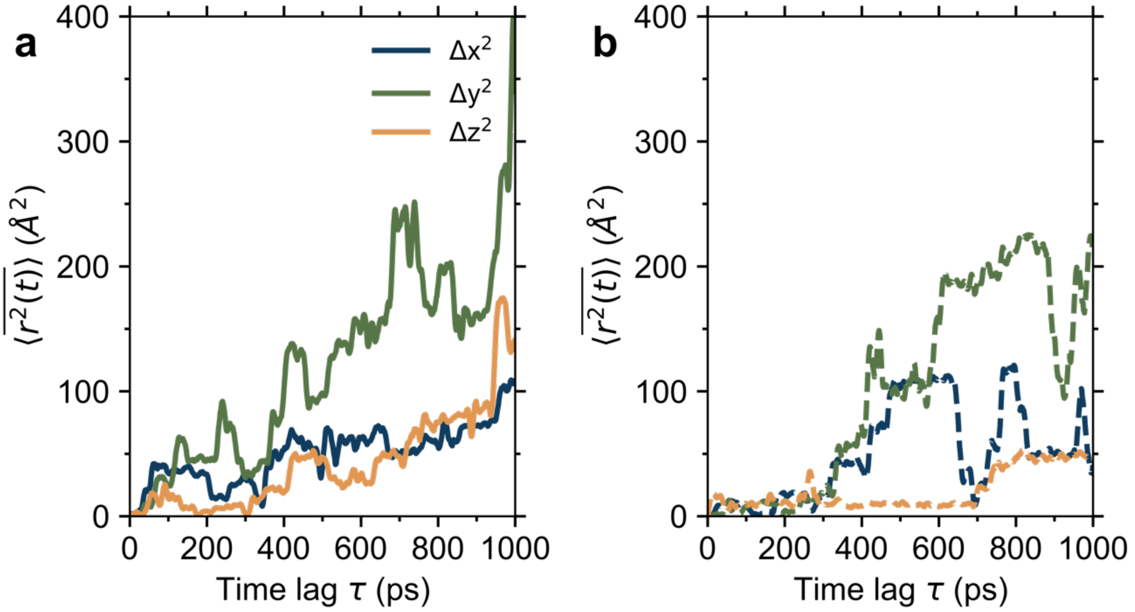

Fig. S17. MSD at 900 K of (**a**) glass and (**b**) interfacial phase in different directions for the glass-ceramic Li_3PS_4. Source data are provided as a Source Data file.

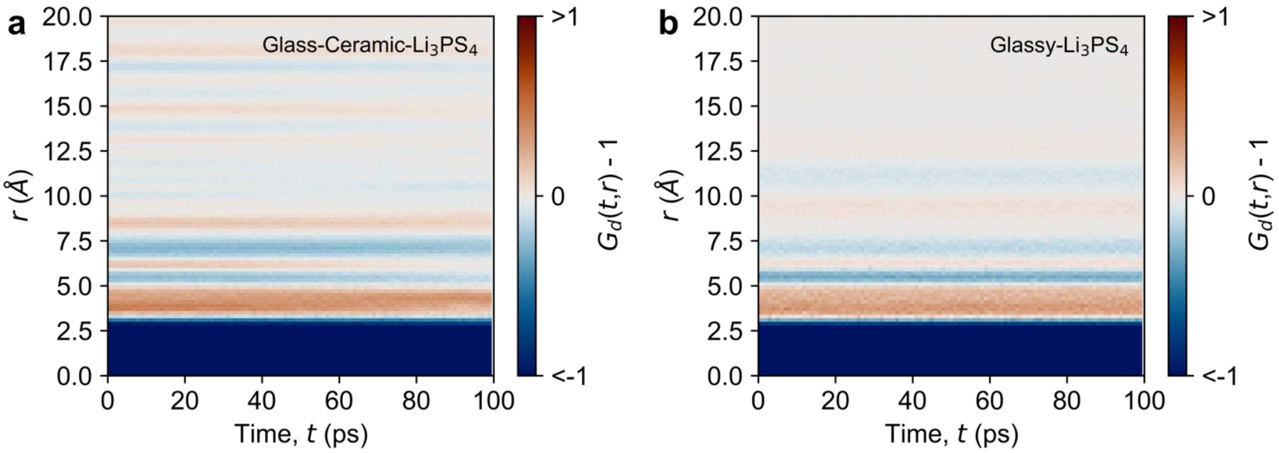

Fig. S18. Distinct-part of van Hove correlation function for glass-ceramic (**a**) and glassy Li_3PS_4 (**b**) at 300 K.

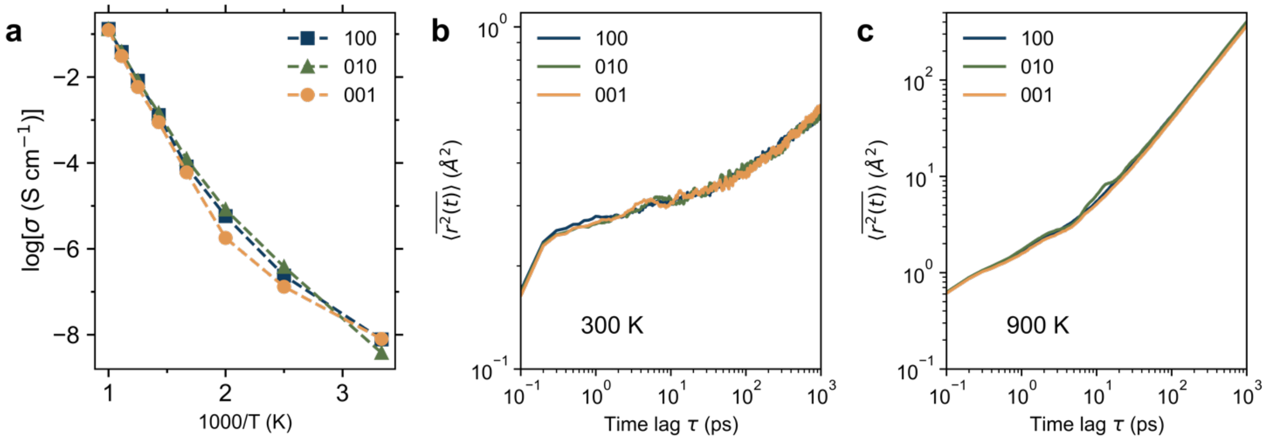

Fig. S19. **a** Temperature dependence of ionic conductivity of glass-ceramic Li_3PS_4. Three different orientations of crystalline phases within the glass-ceramic are considered. **b,c** MSD of glass-ceramic Li_3PS_4 at 300 K (**b**) and 900 K (**c**). Source data are provided as a Source Data file.

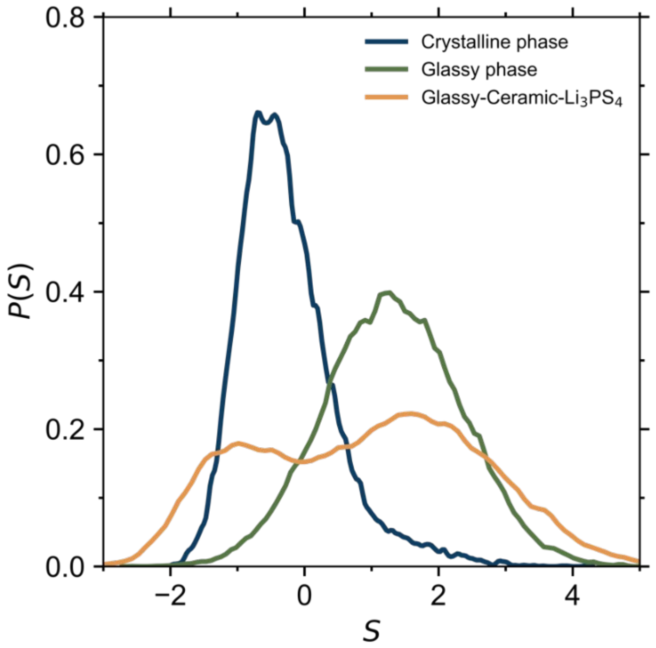

Fig. S20. Distribution of lithium softness *S* for crystalline and glassy phases within the glass-ceramic Li_3PS_4 as well as the average of the glass-ceramic Li_3PS_4 at 300 K. Source data are provided as a Source Data file.

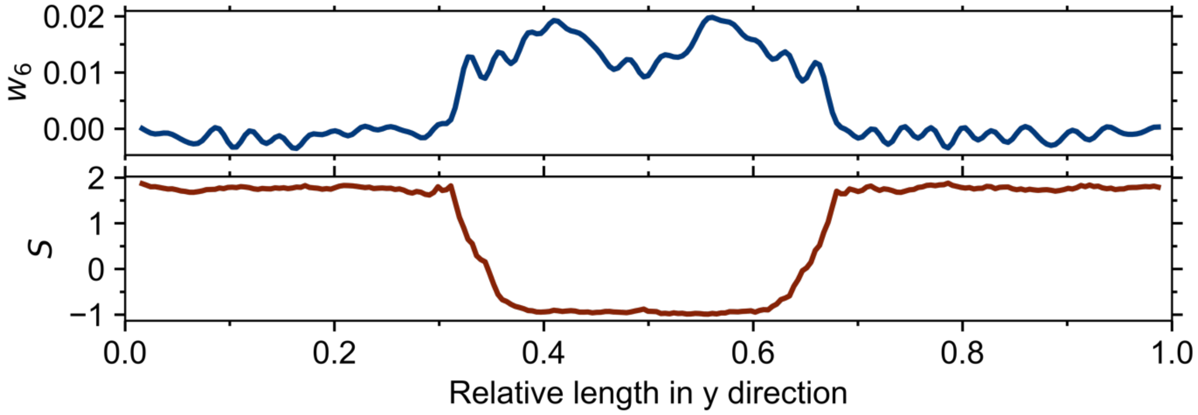

Fig. S21. Profiles of the averaged w_l order parameters (top) and lithium softness S (bottom) along the y-direction in the glass-ceramic Li_3PS_4 configuration at 300 K. Source data are provided as a Source Data file.

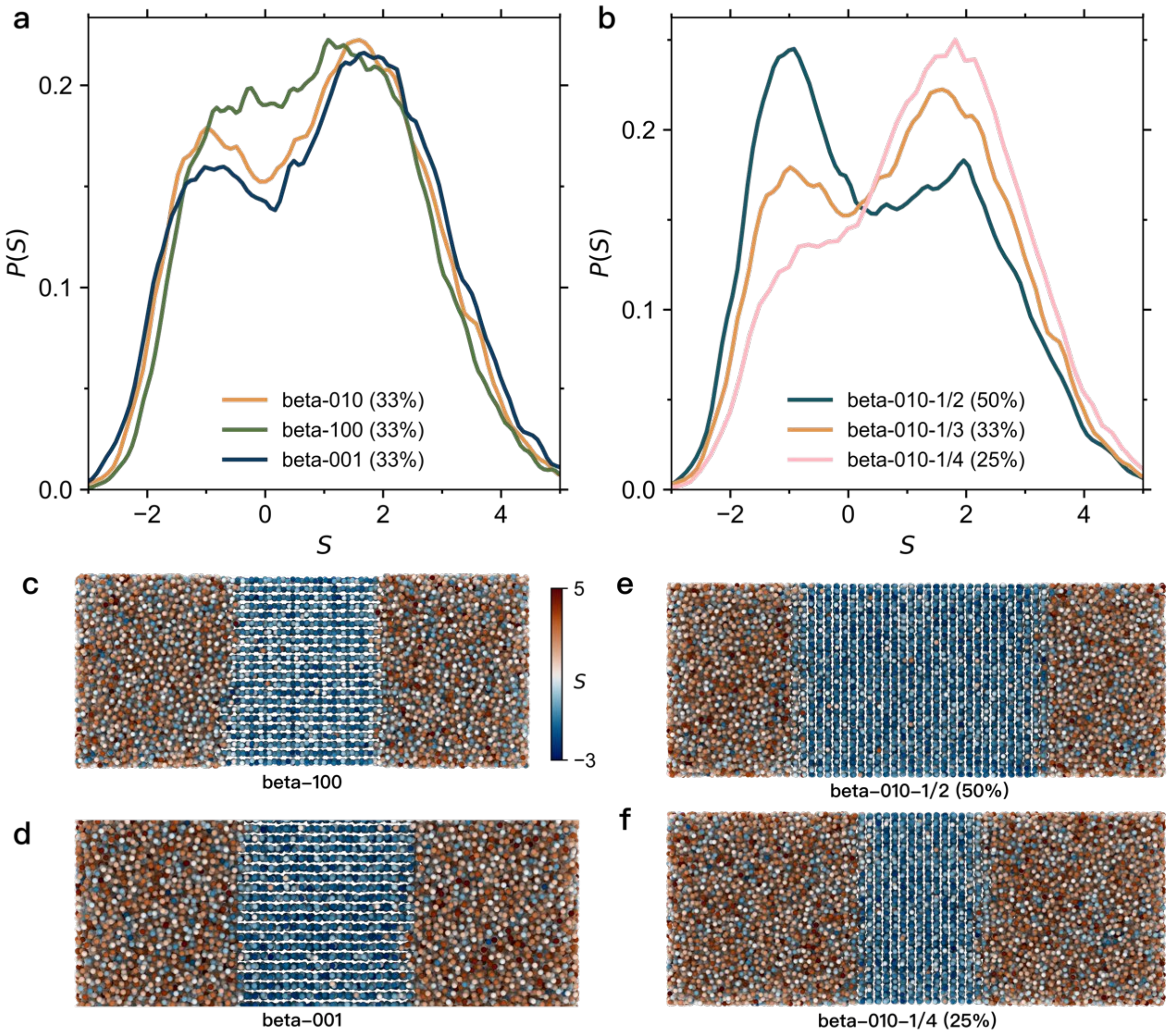

Fig. S22. a,b Distribution of particle softness S for the glass-ceramic Li_3PS_4 with **a** different crystal (β-Li_3PS_4) orientations and **b** varying crystal content. Source data are provided as a Source Data file. **c-f** Corresponding atomic snapshots, where only lithium atoms are presented and colored according to their particle softness S value.

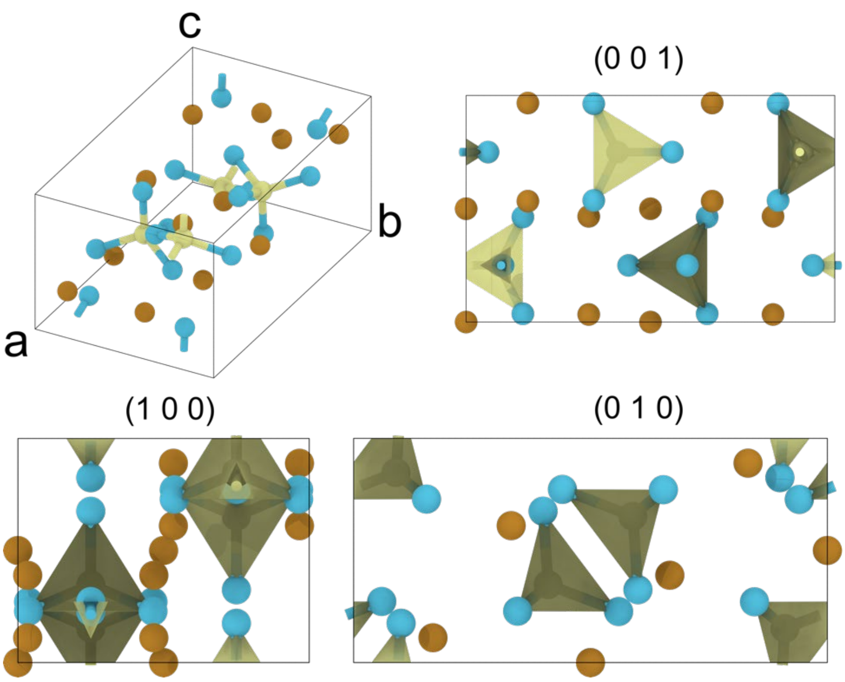

Fig. S23. Visual representation of the β-Li_3PS_4 unit cell, highlighting the crystalline planes of (0 0 1), (1 0 0), and (0 1 0). The space group of β-Li_3PS_4 is *Pnma*. Lattice constants: a = 13.03282 Å, b = 8.01860 Å, c = 6.17177 Å, α = 90, β = 90, γ = 90.

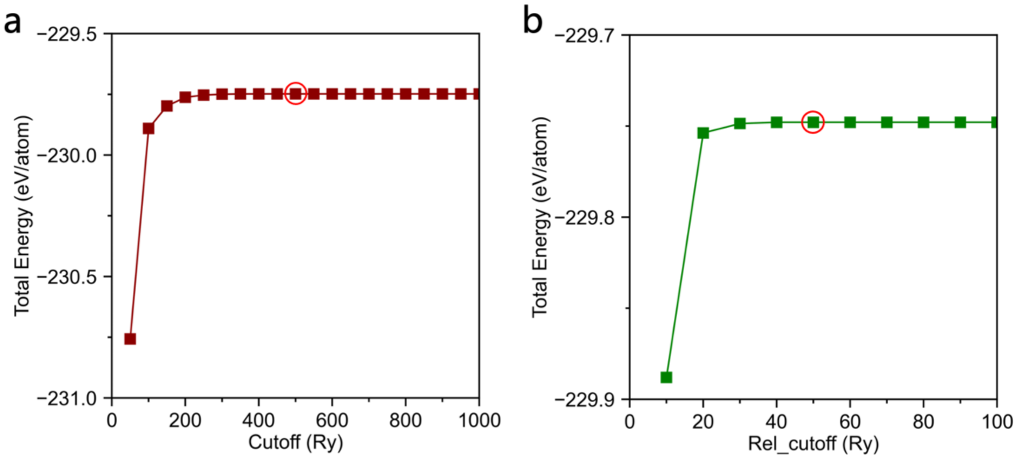

Fig. S24. Dependence of total energy of β-Li_3PS_4 on (a) the plane-wave cutoff for the electronic density and (b) the relative cutoff. The values highlighted in red circles are adopted in this study. Source data are provided as a Source Data file.

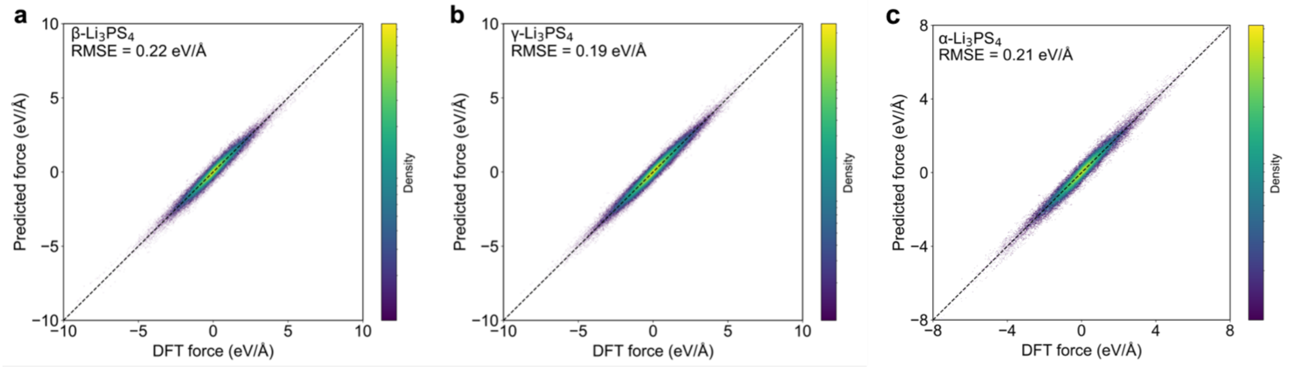

Fig. S25. Comparison of MLIP predicted and DFT calculated atomic forces for **a** β-Li_3PS_4, **b** γ-Li_3PS_4, and **c** α-Li_3PS_4 systems during the melting process. Source data are provided as a Source Data file.

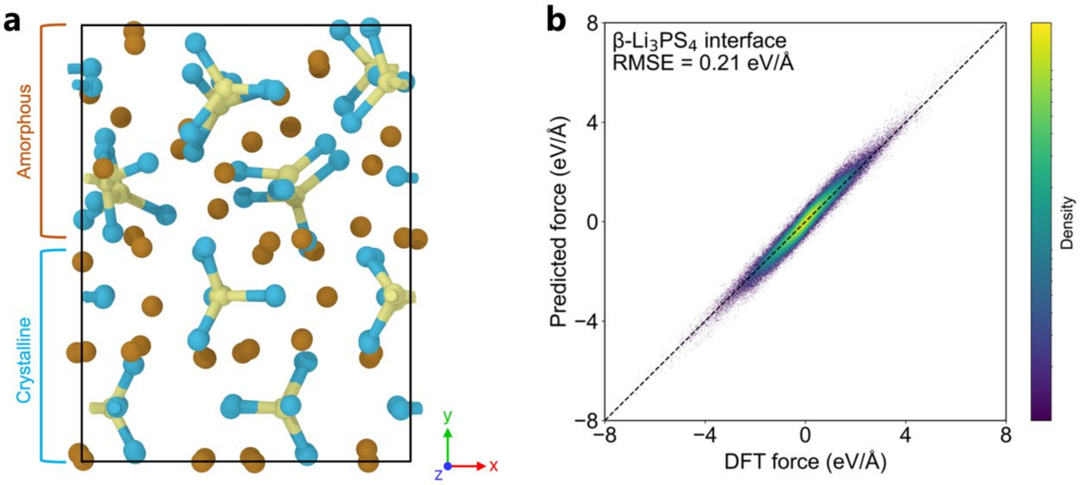

Fig. S26. a Atomic structure of the interface between the β-Li_3PS_4 crystal and amorphous structures used for *ab initio* MD simulations. Lithium atoms are colored in brown, sulfur atoms in blue, and phosphorus atoms in yellow. **b** Comparison of atomic forces from MLIP predictions and DFT calculations for the crystal-amorphous structure of β-Li_3PS_4. Here, the MLIP was trained without the interfacial structure in the dataset. Source data are provided as a Source Data file.

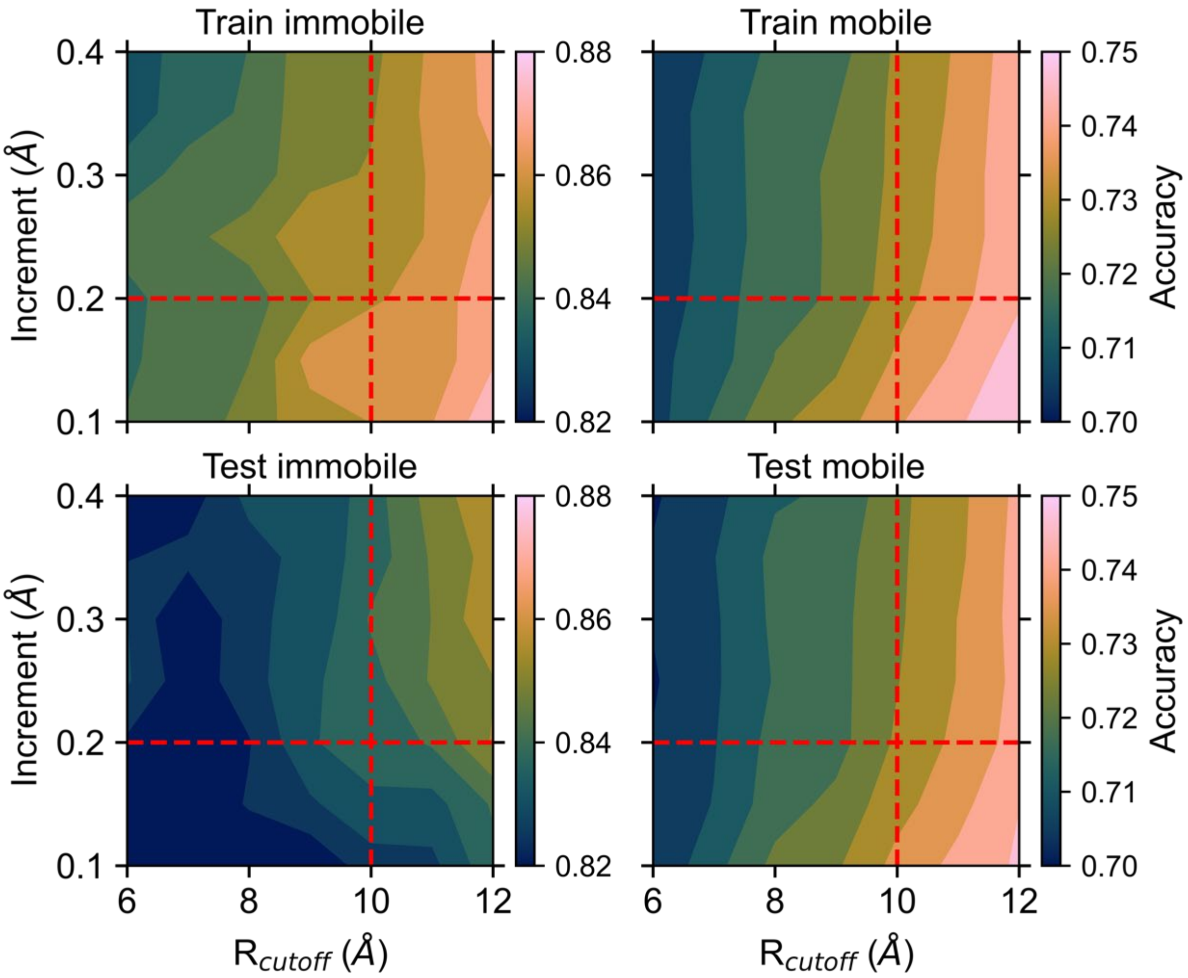

Fig. S27. Classification accuracy of the logistic regression model as a function of the *dr* and R_{cutoff} values of the radial order parameters to discriminate the mobile and immobile lithium atoms.

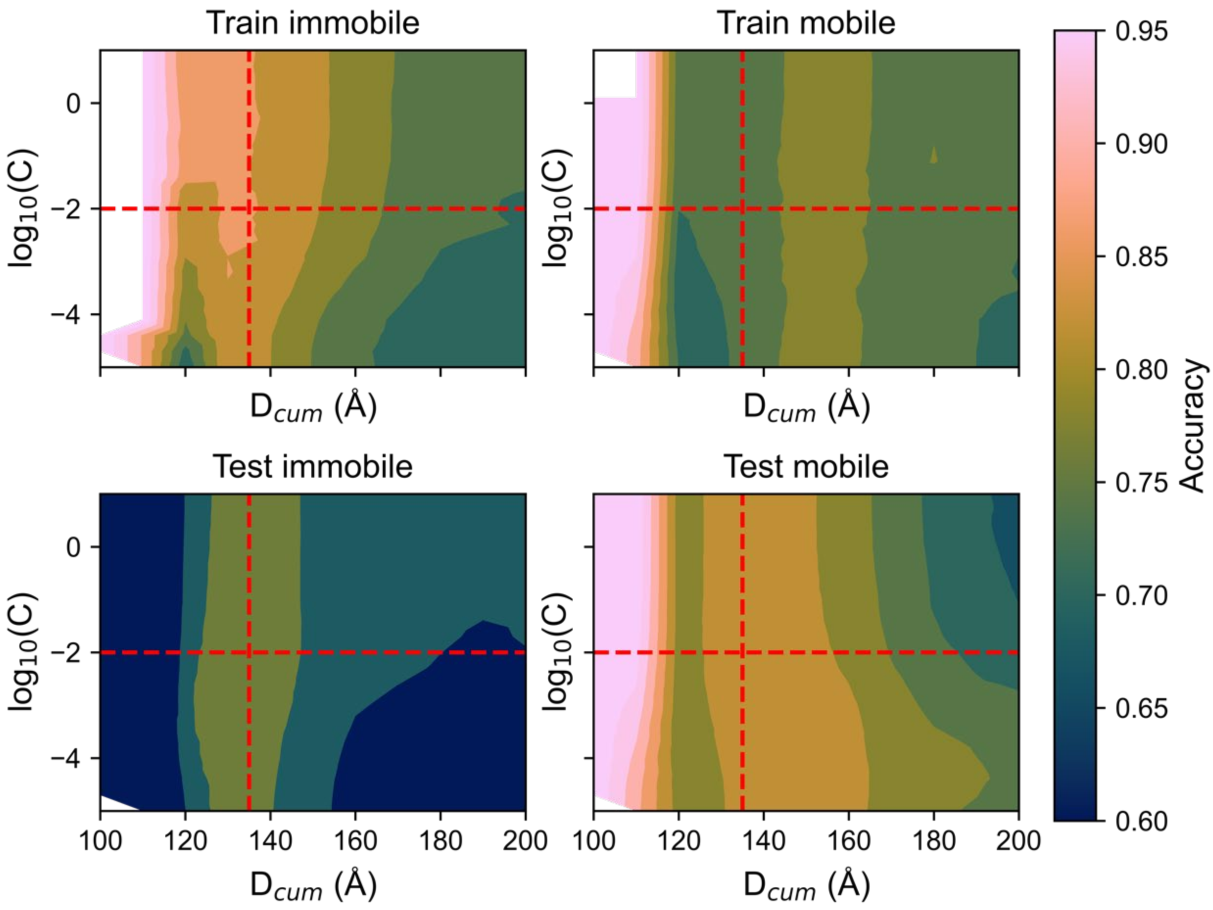

Fig. S28. Classification accuracy of the logistic regression model as a function of the regularization parameter *C* and cumulative non-affine displacement threshold D_c to discriminate the mobile and immobile lithium atoms.

Supplementary Tables

Table S1. Lattice parameters, room temperature ionic conductivity (σ_{RT}), and conduction activation energy (E_a) of Li_3PS_4 electrolytes. Data are included from this work as well as literature, covering both experimental results (based on x-ray diffraction) as well as simulation results using different methods. The abbreviations used in the table are as follows: Exp. (experiment), DFT (density functional theory), CMD (classical molecular dynamics), and MTP (moment tensor potential).

**Li_3PS_4**	**Lattice parameters**			**σ_{RT} (S cm^{-1})**	**E_a (eV)**
	a	***b***	***c***		
β-Li_3PS_4 (this work)	13.01	8.34	6.22	4.8×10^{-6}	0.49
Exp.[3]	12.82	8.22	6.12	8.9×10^{-7}	0.48[4]
Exp.[5]	12.98	8.04	6.13		
PBE[6]	13.07	8.13	6.26		
PBE[7]	13.02	8.17	6.25	6×10^{-5}	0.49
PBE (this work)	13.03	8.02	6.17		
PBEsol (this work)	12.85	7.93	6.10		
PBEsol[8]				7.7×10^{-4}	0.38
PBE0[8]				8.7×10^{-6}	0.62
CMD[9]				$\sim 10^{-2}$	<0.2
MTP[10]	13.06	8.12	6.26	7.4×10^{-5}	0.29
Li_3PS_4 glass (this work)				1.3×10^{-4}	
Exp.[11]				1.8×10^{-4}	
Li_3PS_4 glass-ceramic (this work)				2.2×10^{-4}	
Exp.[11]				2.8×10^{-4}	

Table S2. Details of the initial data set obtained from the *ab initio* MD simulations and used to train the MLIP.

System	Composition	Atom number	Number of configurations
β-Li_3PS_4	Li24P8S32	64	1000
γ-Li_3PS_4	Li48P16S64	128	1000
$Li_2P_2S_6$	Li16P16S48	80	1000
Hexagonal Li_2PS_3	Li32P16S48	96	1000
Orthorhombic Li_2PS_3	Li32P16S48	96	1000
Li_2S	Li64S32	96	1000
Li_3P	Li48P16	64	1000
$Li_4P_2S_6$	Li32P16S48	96	1000
$Li_7P_3S_{11}$	Li28P12S44	84	1000
Li_7PS_6	Li28P4S24	56	1000
$Li_{48}P_{16}S_{61}$	Li48P16S61	125	1000
P_2S_5	P8S20	28	1000
P_4S_3	P32S24	56	1000
67Li_2S-33P_2S_5	Li82P40S138	260	1000
70Li_2S-30P_2S_5	Li82P38S133	253	1000
75Li_2S-25P_2S_5	Li91P35S129	255	1000
80Li_2S-20P_2S_5	Li92P34S128	254	1000
Li	Li54	54	1000
P	P48	48	1000
S	S32	32	1000

Table S3. Details of the explored data set obtained from the single energy calculation using CP2K.

Iteration	System	Composition	Atom number	Conditions	Number of configurations
1	67Li2S-33P2S5	Li82P40S138	260	Temp(K):	579
	70Li2S-30P2S5	Li82P38S133	253	[500,800,1000]	425
	75Li2S-25P2S5	Li91P35S129	255	Press(bar):[0,50]	159
2	67Li2S-33P2S5	Li82P40S138	260	Temp(K):	2344
	70Li2S-30P2S5	Li82P38S133	253	[500,800,1000]	2309
	75Li2S-25P2S5	Li91P35S129	255	Press(bar):[10,1000]	802
3	67Li2S-33P2S5	Li82P40S138	260	Temp(K):	96
	70Li2S-30P2S5	Li82P38S133	253	[500,800,1000]	63
	75Li2S-25P2S5	Li91P35S129	255	Press(bar):[5000,10000]	23
4	67Li2S-33P2S5	Li82P40S138	260	Temp(K):	1404
	70Li2S-30P2S5	Li82P38S133	253	[900,1200,1500]	1459
	75Li2S-25P2S5	Li91P35S129	255	Press(bar):[0,50]	909
5	67Li2S-33P2S5	Li82P40S138	260	Temp(K):	1492
	70Li2S-30P2S5	Li82P38S133	253	[900,1200,1500]	1495
	75Li2S-25P2S5	Li91P35S129	255	Press(bar):[10,1000]	866
6	67Li2S-33P2S5	Li82P40S138	260	Temp(K):	1492
	70Li2S-30P2S5	Li82P38S133	253	[900,1200,1500]	1489
	75Li2S-25P2S5	Li91P35S129	255	Press(bar):[5000,10000]	756
7	67Li2S-33P2S5	Li82P40S138	260	Temp(K):	1498
	70Li2S-30P2S5	Li82P38S133	253	[900,1200,1500]	1490
	75Li2S-25P2S5	Li91P35S129	255	Press(bar):[0,50]	1491
8	67Li2S-33P2S5	Li82P40S138	260	Temp(K):	1498
	70Li2S-30P2S5	Li82P38S133	253	[1400,1600,1800]	1495
	75Li2S-25P2S5	Li91P35S129	255	Press(bar):[10,1000]	1490
9	67Li2S-33P2S5	Li82P40S138	260	Temp(K):	1497
	70Li2S-30P2S5	Li82P38S133	253	[1400,1600,1800]	1498
	75Li2S-25P2S5	Li91P35S129	255	Press(bar):[5000,10000]	1491
10	67Li2S-33P2S5	Li82P40S138	260	Temp(K):	1490
	70Li2S-30P2S5	Li82P38S133	253	[400,800,1200]	1497
	75Li2S-25P2S5	Li91P35S129	255	Press(bar):[100]	1491
11	67Li2S-33P2S5	Li82P40S138	260	Temp(K):	1488
	70Li2S-30P2S5	Li82P38S133	253	[400,800,1200]	1489
	75Li2S-25P2S5	Li91P35S129	255	Press(bar):[2000]	1250

Supplementary References

1. Ohara, K. *et al.* Structural and electronic features of binary Li_2S-P_2S_5 glasses. *Sci. Rep.* **6**, 21302 (2016).
2. Zhou, R., Luo, K., Martin, S. W. & An, Q. Insights into Lithium Sulfide Glass Electrolyte Structures and Ionic Conductivity via Machine Learning Force Field Simulations. *ACS Appl. Mater. Interfaces* **16**, 18874–18887 (2024).
3. Homma, K. *et al.* Crystal structure and phase transitions of the lithium ionic conductor Li_3PS_4. *Solid State Ionics* **182**, 53–58 (2011).
4. Tachez, M., Malugani, J.-P., Mercier, R. & Robert, G. Ionic conductivity of and phase transition in lithium thiophosphate Li_3PS_4. *Solid State Ionics* **14**, 181–185 (1984).
5. Chen, Y. *et al.* Correlation of anisotropy and directional conduction in β-Li_3PS_4 fast Li+ conductor. *Appl. Phys. Lett.* **107**, 013904 (2015).
6. Holekevi Chandrappa, M. L., Qi, J., Chen, C., Banerjee, S. & Ong, S. P. Thermodynamics and Kinetics of the Cathode–Electrolyte Interface in All-Solid-State Li–S Batteries. *J. Am. Chem. Soc.* (2022).
7. Žguns, P. & Yildiz, B. Strain Sensitivity of Li-ion Conductivity in β-Li_3PS_4 Solid Electrolyte. *PRX Energy* **1**, 023003 (2022).
8. Gigli, L., Tisi, D., Grasselli, F. & Ceriotti, M. Mechanism of Charge Transport in Lithium Thiophosphate. *Chem. Mater.* **36**, 1482–1496 (2024).
9. Ariga, S., Ohkubo, T., Urata, S., Imamura, Y. & Taniguchi, T. A new universal force-field for the Li_2S–P_2S_5 system. *Phys. Chem. Chem. Phys.* **24**, 2567–2581 (2022).
10. Jalem, R., Chandrappa, M. L. H., Qi, J., Tateyama, Y. & Ong, S. P. Lithium dynamics at grain boundaries of β-Li3PS4 solid electrolyte. *Energy Adv.* **2**, 2029–2041 (2023).
11. Hayashi, A., Hama, S., Minami, T. & Tatsumisago, M. Formation of superionic crystals from mechanically milled Li_2S–P_2S_5 glasses. *Electrochem. Commun.* **5**, 111–114 (2003).
